# Editorial: Dietary habits in liver health and disease: preclinical and clinical studies

**DOI:** 10.3389/fnut.2025.1670605

**Published:** 2025-08-18

**Authors:** Evelyn Nunes Goulart da Silva Pereira, Caroline Fernandes-Santos

**Affiliations:** ^1^Laboratory of Clinical and Experimental Physiopathology, Oswaldo Cruz Institute, Oswaldo Cruz Foundation, Rio de Janeiro, Brazil; ^2^Department of Basic Sciences, Federal Fluminense University, Nova Friburgo, Brazil

**Keywords:** dietary inflammatory index, ultra-processed foods, insulin resistance markers, liver fibrosis, nutritional interventions

The intricate relationship between dietary habits and liver health has become a rapidly growing field of research, especially in light of the worldwide increase in chronic liver disease (CLD) (1). The liver is the central organ responsible for regulating metabolism, detoxification, and modulating the immune system and is also susceptible to nutrients (2). Understanding how diet influences liver function and pathology is crucial for developing effective strategies to prevent and treat liver disease. This research theme combines preclinical and clinical investigations that examine the impact of dietary patterns, nutrients, and metabolic indicators on liver health, offering new insights for researchers and clinicians.

The increasing prevalence of non-alcoholic fatty liver disease (NAFLD) worldwide, now often referred to as metabolic dysfunction-associated steatotic liver disease (MASLD), underscores the critical role of diet in liver pathology. NAFLD is driven by increasing obesity and diabetes and can lead to more serious conditions such as fibrosis, cirrhosis, and hepatocellular carcinoma (3). The economic impact is substantial, necessitating a coordinated global effort to address the growing burden of CLD (4).

Recent research has focused on identifying specific dietary components and patterns that contribute to the development of liver disease. For example, the impact of pro-inflammatory diets on the risk of CLD has been studied in detail. A comprehensive analysis of data from the UK Biobank cohort revealed a significant association between a higher Dietary Inflammatory Index (DII), which indicates a more inflammatory dietary pattern, and an increased risk of CLD. This robust finding, consistent with various demographic and lifestyle factors, strongly suggests that adopting anti-inflammatory dietary patterns may be a critical strategy to mitigate the global burden of CLD (Pan et al.).

In addition to general dietary patterns, the role of specific food categories, such as ultra-processed foods (UPF), has received increasing attention. A cross-sectional analysis of 4,992 adults from the National Health and Nutrition Examination Survey (NHANES) 2017–2020 cycle showed that higher UPF consumption was significantly associated with increased liver fat accumulation, as measured by the controlled attenuation parameter (CAP). These results underscore the deleterious impact of UPF on liver steatosis, particularly in individuals who are overweight or have increased waist circumference (Song et al.).

The interaction between diet, metabolic health, and liver function extends deep into the realm of insulin resistance (IR). IR is a well-established factor in the pathogenesis of NAFLD and its progression to liver fibrosis. Yang et al. confirm a significant association between various IR indexes and liver fibrosis in NAFLD patients. In particular, the triglyceride glucose-waist to height ratio (TyG-WHtR) has been shown to be a prominent predictor of liver fibrosis, even after adjustment for covariates. This work highlights the potential of incorporating IR indexes into routine clinical practice for early risk assessment and timely interventions to prevent the progression of fibrosis.

In addition, the broader concept of cardiovascular health, as the Life's Essential 8 (LE8) construct from the American Heart Association, has been linked to liver function. A cross-sectional study using data from the NHANES 2007–2018 cycle revealed that higher LE8 scores are associated with better liver function, particularly with lower levels of liver enzymes, including ALT, ALT/AST ratio, ALP, and GGT (Liang et al.). This association exhibits non-linear patterns and is more pronounced in younger individuals. These findings suggest that comprehensive interventions to improve cardiovascular health, which include a balanced diet, regular physical activity, and other lifestyle factors, may also benefit liver health.

The impact of nutritional interventions also extends to specific clinical contexts, such as postoperative care for patients with colorectal cancer (CRC). Malnutrition is common in CRC patients, hindering recovery and increasing the risk of complications. Research has shown that early postoperative administration of dietary fiber significantly improves immune function, reduces inflammation, and improves the nutritional status in CRC patients (Ji et al.). This evidence highlights the importance of tailoring nutritional support strategies, including dietary fiber, to achieve optimal patient outcomes. Machine learning models have also been successfully used to predict the impact of dietary fiber on immune function and inflammatory responses. Important predictors, such as procalcitonin (PCT), prealbumin (PAB), albumin (ALB), and interleukin-1 (IL-1), were identified (Ji et al.).

Finally, the role of trace elements in liver health, which is influenced by dietary intake and environmental exposure, is also gaining increasing attention. For example, He et al. have examined the association between serum manganese (Mn) levels and NAFLD. The results indicate that higher serum Mn levels are associated with an increased risk of NAFLD, with sex-specific differences in the dose-response relationship. This study emphasizes the importance of further investigating the intricate relationship between trace elements, environmental factors, and CLD pathogenesis, to develop sex-specific prevention strategies.

In summary, there is consistent and converging evidence that provides a substantial amount of relevant new data on the role of diet composition, dietary patterns, and the benefits of dietary interventions to liver health and disease. Despite the extensive literature available on this important topic, the papers published in this Research Topic demonstrate that some gaps still exist in various aspects of the complex area of diet's influence on liver function, which remain to be clarified and better understood. After reading this volume, readers will have a clearer understanding of topics such as the impact of inflammatory diets, the role of specific sugars, the importance of IR markers, the wide-ranging benefits of cardiovascular health metrics, and the nuanced effects of trace minerals, reinforcing the understanding that dietary habits are a cornerstone in the prevention and treatment of liver disease.
